# Dermatology Resident Training on Depression Screening: A Cross-Sectional Survey

**DOI:** 10.7759/cureus.8861

**Published:** 2020-06-27

**Authors:** Kaitlyn L Streight, Harry Dao, Soo Jung Kim

**Affiliations:** 1 Dermatology, Baylor College of Medicine, Houston, USA; 2 Dermatology, Loma Linda University Health, Loma Linda, USA

**Keywords:** dermatology residency curriculum, dermatology education, depression screening in dermatology, mental health comorbidities, resident education, quality improvement research, survey research

## Abstract

Background

Many dermatologic diseases are implicated in the development of depression. Currently, there is no literature addressing the extent of dermatology residency training on depression screening.

Objective

Our study aimed to determine the extent of dermatology residency training on depression screening to potentially improve education in this area.

Methods

We designed a 12-question survey to assess the level of resident training and comfort in depression screening for patients with acne vulgaris, atopic dermatitis, psoriasis, hidradenitis suppurativa, and skin cancer. Fifty-six residents completed the survey, and data for each question was analysed in aggregate.

Results

Participants found depression screening most important for patients with acne vulgaris, psoriasis, and hidradenitis suppurativa (p<0.0001, 95% CI). Ratings of confidence in screening were similar across all conditions. Most residents reported only occasional screening in the setting of these diseases. Sixty-four percent of participants stated that education on depression screening is not included as part of their curriculum or clinical practice, while 23% were uncertain.

Conclusions

Our results suggest a lack in relevant training during residency, warranting the inclusion of education on depression screening into the dermatology residency curriculum to facilitate better recognition of the mental health comorbidities of dermatologic diseases.

## Introduction

Psychiatric disturbances have been reported in at least 30% of patients with dermatologic disorders [1]. Common cutaneous conditions implicated in the development of depression include acne vulgaris, atopic dermatitis, psoriasis, and skin cancers [2-4]. Others associated with an increased risk include vitiligo, alopecia areata, ichthyosis, and hidradenitis suppurativa [4,5]. Currently, there is no literature discussing the level of dermatology residency training on depression screening. This study aimed to determine the extent of dermatology residency training on depression screening and to potentially improve education in this area.

## Materials and methods

We created an anonymous 12-question survey to assess the level of resident training and comfort in depression screening for patients with acne vulgaris, atopic dermatitis, psoriasis, hidradenitis suppurativa, and skin cancer (see the Appendices). Our study was granted approval by the Institutional Review Board at Baylor College of Medicine (Houston, TX) in July 2019, and invitations to complete the survey were emailed to members of the Association of Professors of Dermatology electronic mailing list. Membership to this list requires that individuals are from dermatology departments at accredited medical schools or colleges of osteopathic medicine. These recipients were asked to forward our survey invitation to their residents.

## Results

Fifty-six residents from residency programs in all regions of the United States completed the survey, including 27 post graduate year (PGY)-2s, 14 PGY-3s, 12 PGY-4s, and three fellows. Results are presented in Tables 1, 2. Of the five diseases, participants found depression screening most important for patients with hidradenitis suppurativa, with an average rating of 8.3 out of 10 (Table 1). Respondents found screening least important for patients with skin cancer, with an average rating of 4.9 (Table 1). These results were found to be statistically significant upon analysis of variance (ANOVA), with a p-value less than 0.0001. Ratings of confidence in screening were similar across all conditions, with averages ranging from 5.8 to 6.7 (Table 1). Most respondents indicated that they sometimes screen for depression in patients with acne vulgaris, atopic dermatitis, psoriasis, and hidradenitis suppurativa, while the majority reported that they never screen patients with skin cancer (Table 2).

**Table 1:** Dermatology resident ratings of importance and confidence in depression screening The table shows average dermatology resident rating of the importance of depression screening in patients, compared with average confidence in screening, on a scale from 1 to 10. ANOVA, analysis of variance.

Dermatologic disease	Mean perception of importance (SD)	Mean perception of confidence (SD)
Acne vulgaris	7.0 (1.71)	6.5 (2.39)
Atopic dermatitis	6.7 (1.70)	6.4 (2.45)
Psoriasis	7.1 (1.79)	6.6 (2.44)
Hidradenitis suppurativa	8.3 (1.85)	6.7 (2.41)
Skin cancer	4.9 (1.89)	5.8 (2.54)
Statistical significance on ANOVA (95% CI)	p<0.0001	p=0.405

**Table 2:** Reported frequency of depression screening by dermatology residents

Dermatologic disease	Never (0% of the time)	Sometimes (<50% of the time)	Usually (>50% of the time)	Always (100% of the time)
Acne vulgaris	12 (21%)	36 (64%)	7 (13%)	1 (2%)
Atopic dermatitis	21 (38%)	30 (54%)	5 (9%)	0 (0%)
Psoriasis	18 (32%)	25 (45%)	11 (20%)	2 (4%)
Hidradenitis suppurativa	13 (23%)	24 (43%)	15 (27%)	4 (7%)
Skin cancer	36 (64%)	20 (36%)	0 (0%)	0 (0%)

Sixty-four percent of residents stated that training on methods of depression screening is not included as part of their curriculum or clinical practice, while 23% were uncertain. When asked about barriers to screening, 61% indicated lack of time and 45% indicated discomfort due to lack of training. Others included liability (30%), feelings that depression screening is not the dermatologist’s responsibility (11%), and a lack of resources for depressed patients (4%). For all disorders, most participants who screen listed self-reported symptoms as their primary screening method.

## Discussion

Our results reveal that most dermatology residents find depression screening important, particularly for patients with acne vulgaris, psoriasis, and hidradenitis suppurativa, who often experience chronic debilitating symptoms. This finding aligns with previous studies showing the association of acne vulgaris, psoriasis, and hidradenitis suppurativa with depression [2,3,5]. However, most respondents report a lower level confidence in screening and only occasionally screen patients. A previous study demonstrated that depression screening was less common at visits with dermatologists than non-dermatologists [2]. This could be due to the lack of the relevant training during residency.

The limitations of our study include a low response rate, reflecting limitations in our study design. Our request for members of the Association of Professors of Dermatology electronic mailing list to forward our survey to their residents likely prevented us from inviting all residents to participate, contributing to a lower response rate. The geographic distribution of respondents also potentially represents a limitation in the generalizability of the results given the small sample size. We thus encourage future studies to use an alternative method of recruiting residents to increase the sample size. 

To our knowledge, this is the first study to assess provider education on the methods of depression screening in the dermatologic setting. Although larger studies and further collaboration with psychiatrists are needed, our study suggests that improved dermatology residency training on depression screening is warranted to facilitate better recognition of the mental health comorbidities of dermatologic disease, and therefore, implementation of subsequent action plans for proper referral.

## Conclusions

Our results reveal that most dermatology residents find depression screening important, but report a lower level of confidence in screening and only occasionally screen patients. Most residents indicated that education on methods of depression screening is not included in their residency curriculum, highlighting a lack of training during residency. These findings suggest a need for collaboration with psychiatrists to improve education on depression screening in the dermatology residency curriculum and facilitate better recognition of the mental health comorbidities of dermatologic disease.

## Appendices

Depression Screening in Dermatologic Patients: Resident Survey

1. What year of dermatology residency training are you currently in?

a) PGY2

b) PGY3

c) PGY4

d) Fellowship

e) Other (please specify): _______________

2. In what state is your residency program located? ____________________________

3. Please rate your perception of the importance of depression screening in patients with the following skin diseases on a scale from 1 (least important) to 10 (most important).

**Table 3:** Question 3 matrix on survey

Dermatologic disease										
Acne vulgaris	1	2	3	4	5	6	7	8	9	10
Atopic dermatitis	1	2	3	4	5	6	7	8	9	10
Psoriasis	1	2	3	4	5	6	7	8	9	10
Hidradenitis suppurativa	1	2	3	4	5	6	7	8	9	10
Skin cancer	1	2	3	4	5	6	7	8	9	10

4. Please rate your overall confidence in screening patients with the following skin diseases for depression on a scale from 1 (least confident) to 10 (most confident).

**Table 4:** Question 4 matrix on survey

Dermatologic disease										
Acne vulgaris	1	2	3	4	5	6	7	8	9	10
Atopic dermatitis	1	2	3	4	5	6	7	8	9	10
Psoriasis	1	2	3	4	5	6	7	8	9	10
Hidradenitis suppurativa	1	2	3	4	5	6	7	8	9	10
Skin cancer	1	2	3	4	5	6	7	8	9	10

5. Is training on the various tools of depression screening in dermatologic patients included as part of your curriculum or part of your standard clinical practice?

a) Yes

b) No

c) Unsure

6. How often do you screen your patients with the following skin diseases for depression?

**Table 5:** Question 6 matrix on survey

	Never (0% of the time)	Sometimes (<50% of the time)	Usually (≥50% of the time)	Always (100% of the time)
Acne vulgaris				
Atopic dermatitis				
Psoriasis				
Hidradenitis suppurativa				
Skin cancer				

7. If you screen your patients with acne vulgaris for depression, what screening tool do you primarily use?

a) Self-reported symptoms

b) Symptom Checklist-90 (SCL-90)

c) Patient Health Questionnaire-2 Scale (PHQ-2)

d) Patient Health Questionnaire-9 Scale (PHQ-9)

e) Not applicable/do not screen for depression in these patients

f) Other (please specify): ___________________

8. If you screen your patients with atopic dermatitis for depression, what screening tool do you primarily use?

a) Self-reported symptoms

b) Children’s Dermatology Life Quality Index (CDLQI)

c) Children’s Depression Inventory (CDI)

d) Multidimensional Anxiety Scale for Children (MASC)

e) Not applicable/do not screen for depression in these patients

f) Other (please specify): ___________________

9. If you screen your patients with psoriasis for depression, what screening tool do you primarily use?

a) Self-reported symptoms

b) Patient Health Questionnaire-2 Scale (PHQ-2)

c) Hospital Anxiety and Depression Scale (HADS)

d) Mental Health Inventory-5 Scale (MHI-5)

e) Not applicable/do not screen for depression in these patients

f) Other (please specify): ___________________

10. If you screen your patients with hidradenitis suppurativa for depression, what screening tool do you primarily use?

a) Self-reported symptoms

b) Beck Depression Inventory (BDI)

c) Major Depression Inventory (MDI)

d) Patient Health Questionnaire-9 Scale (PHQ-9)

e) Not applicable/do not screen for depression in these patients

f) Other (please specify): ___________________

11. If you screen your patients with skin cancer for signs of depression, what screening tool do you primarily use?

a) Self-reported symptoms

b) Patient Health Questionnaire-2 Scale (PHQ-2)

c) EORTC Quality of Life Questionnaire (EORTC QLQ 30)

d) Not applicable/do not screen for depression in these patients

e) Other (please specify): ___________________

12. What barriers prevent you from screening patients with these diseases for depression? (Check all that apply)

a) I do not feel that depression screening is important in these patients

b) I do not feel that depression screening in these patients is my responsibility

c) I do not feel comfortable screening these patients for depression due to lack of training in depression screening tools

d) Depression screening in these patients takes too much time

e) I am worried about the liability associated with depression screening in these patients

f) Other (please specify): _______________
